# Bushmeat Consumption and the Risk of Zoonotic Tick‐Borne Pathogen Infections in Ghana: An Increasing Risk to Public Health

**DOI:** 10.1002/puh2.70096

**Published:** 2025-07-30

**Authors:** Christopher Nii Laryea Tawiah‐Mensah, Danielle Ladzekpo, Seth Offei Addo

**Affiliations:** ^1^ Parasitology Department Noguchi Memorial Institute for Medical Research, University of Ghana Accra Ghana

**Keywords:** bushmeat | Ghana | public health | ticks | zoonoses

## Abstract

Consuming bushmeat is a widely accepted tradition in Ghana and other West African countries, where it is a vital source of income and protein for many rural populations. However, there are considerable risks associated with this behavior, especially when it comes to zoonotic tick‐borne infections. Zoonotic tick‐borne pathogens are common in wildlife and can spread to people when they handle or eat inadequately prepared bushmeat. This article addresses the growing threat of zoonotic tick‐borne infections associated with consuming bushmeat in Ghana, which is made worse by factors including deforestation, climate change, and more frequent interactions between people and wildlife. Public health risks are heightened due to limited knowledge and awareness of tick‐borne infections and inadequate food safety standards, particularly in rural areas where consuming bushmeat is widespread. To mitigate the risks of zoonotic tick‐borne pathogen transmission, this perspective advocates for urgent public health interventions, including stricter regulations on bushmeat handling and sales, enhanced wildlife surveillance, and increased public health education on the dangers of zoonotic diseases.

## Graphical abstract

• Consuming bushmeat comes with the risk of zoonotic tick‐borne pathogen infection.

• To protect public health, there is a need for regulations on bushmeat handling and sales, frequent surveillance, and increased education.

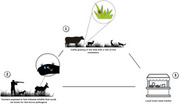

## 1 Introduction

Majority of emerging human pathogens are zoonotic, meaning they spread from animal to human and vice versa [1]. It has been reported that almost two‐thirds of emerging infectious diseases are caused by zoonoses, and most zoonoses originate from wildlife [2]. Furthermore, it has been suggested that human‐wildlife interactions are increasing due to habitat fragmentation and deforestation, which heightens the likelihood of zoonotic pathogens infecting human populations [3]. Despite the risk posed by interactions with wildlife, bushmeat, which is the meat of wild animals hunted for food, remains a popular food source in many West African communities, where they are considered a delicacy [4]. In addition to being a vital source of nutrition, particularly in rural regions [5], the bushmeat trade exposes individuals to zoonotic infections through frequent interaction with wildlife [6]. Zoonotic tick‐borne pathogens can spill over from wildlife to human populations through the trade and consumption of bushmeat. The majority of ticks are ectoparasites of wild animals, with about 10% of tick species feeding on domestic animals [7]. Wild animals are important reservoirs for numerous tick‐borne pathogens, serving as a source of infection for domestic animals and humans through tick infestation [8]. When skinning and slaughtering wild animals, hunters and traders sometimes come into close contact with ticks and could become infected with zoonotic pathogens [9]. Furthermore, after dropping off wildlife, ticks can bite people or infest domestic animals, potentially transmitting zoonotic pathogens [7]. In Ghana, tick species have been reported to infest various wild animals [10, 11]. It is important to note that some of these tick species, including *Amblyomma variegatum* and *Rhipicephalus sanguineus*, have been reported to infest livestock, especially cattle. *A. variegatum* is the predominant tick species infesting cattle in Ghana, and this species has been identified to harbor zoonotic pathogens, including *Rickettsia africae*, *Coxiella burnetii*, and Crimean–Congo hemorrhagic fever virus (CCHFV) [12, 13, 14]. Because cattle are often sent out to graze, there is an increased chance of interactions with wild animals, leading to the exchange of ticks and tick‐borne pathogens. This is more so as a study in Ghana has reported cattle to be infected with the zoonotic pathogen *Anaplasma capra* [15], which infects humans, livestock, and wild animals worldwide [16, 17, 18]. Grasscutter, a large rodent that is frequently hunted and sold in Ghanaian bushmeat markets, is also host to tick‐borne pathogens [19]. A study has reported *Ixodes aulacodi* infesting sampled grasscutters to be infected with parasites and bacteria [20]. Because *I. aulacodi* is a vector for zoonotic tick‐borne pathogens, there is an increased risk of transmission, especially because efforts have been made to breed grasscutter populations locally using wild‐caught ones [21]. In Ghana, there is limited knowledge of the role of wild animals in the spread of ticks and zoonotic tick‐borne pathogens. With the increasing demand for bushmeat [22], there is an increased risk to public health and a need to adopt preventive measures. This article examines how the consumption of bushmeat contributes to the spread of zoonotic tick‐borne pathogens in Ghana, looks at the consequences for public health, and offers solutions to lower this rising risk.

### 1.1 Bushmeat and the Risk of Zoonotic Tick‐Borne Pathogens in Ghana

**FIGURE 1:**
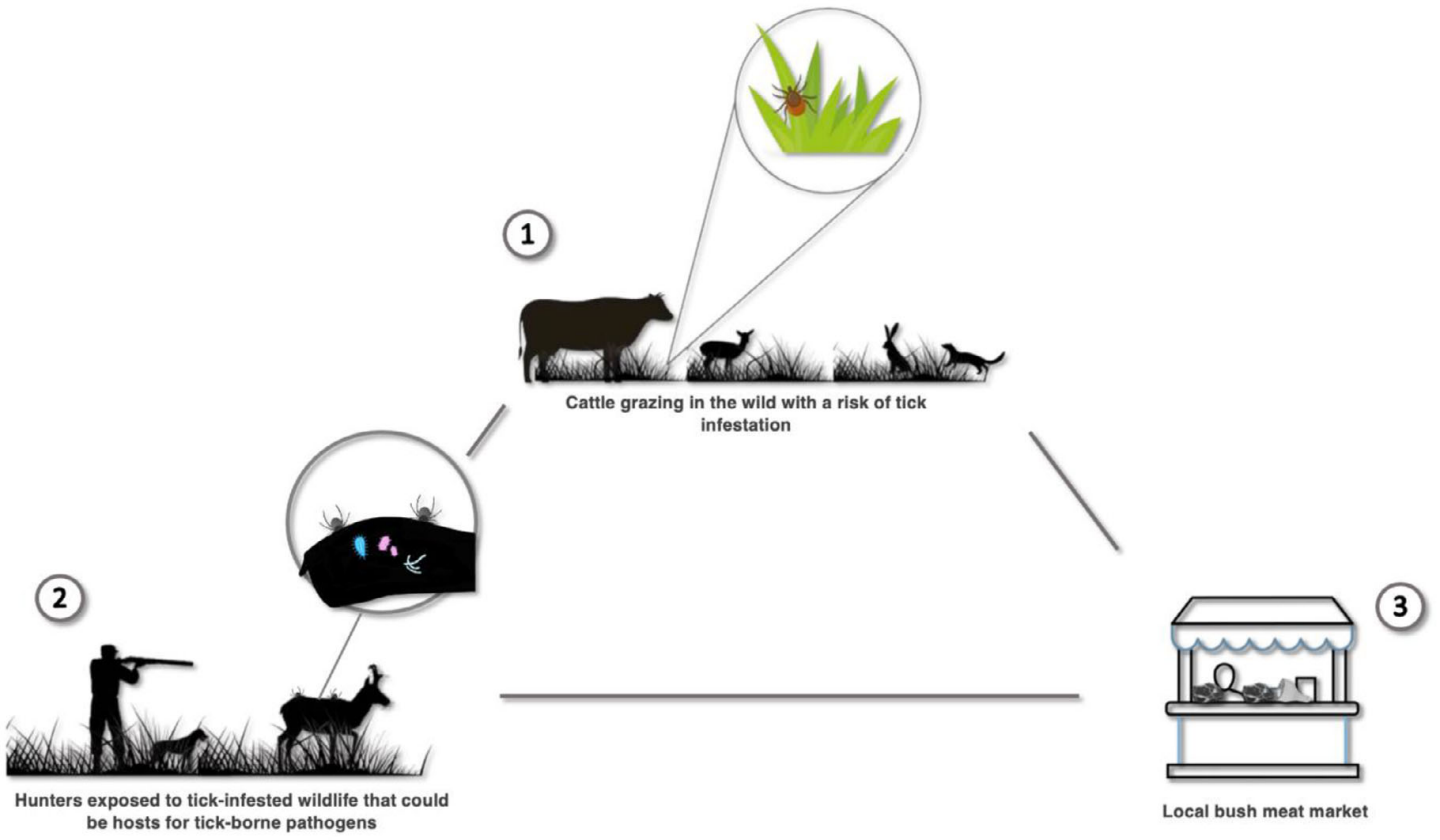
An illustrative view of human‐wildlife‐livestock interaction. (1) Transhumance, the most common method of cattle farming in Ghana, exposes grazing cattle to tick infestation in the wild. (2) During hunting, hunters are exposed to tick bites, and the wild animals they gun down may serve as hosts for tick‐borne pathogens. (3) Bush meat is often undercooked in the bush before being sold to the local bush markets.

Due to the migratory patterns of wildlife, wild animals are responsible for the transmission of numerous zoonotic pathogens both domestically and internationally [23]. Tick exposure can occur when hunters or consumers handle wildlife carcasses, and pathogens can persist on the meat when inappropriate cooking methods are used. As a result of habitat encroachment and the need for bushmeat, human‐wildlife interaction has risen, increasing the risk of zoonotic spillovers [24], including zoonotic tick‐borne pathogens [25]. In various Ghanaian communities, bushmeat has a significant socioeconomic impact. Bushmeat sales occur on the side of the road, frequently in erratic locations, and include a dynamic, mobile, and opportunistic vendor presence. Although hunters are usually found in rural areas, they are increasingly serving urban consumers who can pay higher costs and make stops along highways to acquire animals [26]. Nevertheless, bushmeat preparation and processing are frequently carried out in unregulated environments. Wild animals often consumed as bushmeat in Ghana include, but are not limited to, grasscutter, African giant rat, African civet, bushbuck, red‐flanked duiker, African brush‐tailed porcupine, and royal antelope [10]. It is important to note that wild animals consumed as bushmeat in Ghana have been found infested with diverse tick species [10]. Some of these tick species have been reported to infest livestock and harbor zoonotic pathogens [12, 13, 27, 28]. Because livestock and wild animals in Ghana are infested with similar tick species, there is an increased likelihood of zoonotic pathogen exchange and risk to public health (Figure 1).

### 1.2 Contributing Factors to Increased Risk

In Ghana, land‐use changes and deforestation have changed wildlife habitats, causing animals to seek food and shelter closer to human settlements. Human‐wildlife‐livestock interactions are more common as a result of this habitat overlap. Wildlife displacement and ecosystem disruption have been caused by mining operations, agricultural development, and forest fragmentation [29, 30]. Consequently, ticks that were once confined to wildlife zones now have closer contact with humans and livestock, increasing the risk of zoonotic pathogen transmission. Another significant factor is climate change. The geographic distribution and life cycle of ticks are changing due to climate change, which allows them to thrive in new locations [31, 32]. The lengthening of the tick‐breeding season due to warmer temperatures and altered rainfall patterns is raising the possibility of human‐tick interactions [33]. Because it raises the risk of zoonotic tick‐borne pathogen transmission, this condition is especially worrisome in Ghana's bushmeat‐eating regions. Cultural practices and food security also influence the risk of zoonotic tick‐borne pathogen infections. Bushmeat consumption has been incorporated into the traditional customs of many Ghanaian communities. However, unsustainable hunting methods and heightened exposure to zoonotic infections have resulted from the growing demand for bushmeat, which has been made worse by food instability in rural areas. Food safety procedures are usually insufficient to stop transmission, and hunters and consumers are typically unaware of the health risks associated with ticks and tick‐borne pathogens [34].

### 1.3 Public Health Implications

In Ghana, eating bushmeat poses serious health risks, especially when it comes to zoonotic tick‐borne pathogens. In remote regions with little access to medical care, zoonotic disease epidemics can have catastrophic consequences for local populations. Failure to implement proper precautions during bushmeat handling could result in undetected zoonotic infections and untreated cases of tick‐borne diseases. It is challenging to determine the complete scope of the public health threat caused by zoonotic infections due to the absence of thorough epidemiological data and the underreporting of tick‐borne diseases [35]. This problem calls for an integrated approach that incorporates community education, wildlife conservation initiatives, and public health interventions.

### 1.4 Strategies for Risk Mitigation

Reducing the negative effects of bushmeat consumption on public health requires increasing knowledge of the dangers of zoonotic tick‐borne infections. Education campaigns should focus on rural areas, stressing the dangers of tick bites and the significance of using safe methods for handling and cooking bushmeat [36, 37]. Programs for monitoring wildlife and vectors must be strengthened to discover zoonotic tick‐borne infections early. Areas at high risk of transmission can be identified with the aid of routine monitoring of tick vectors and wildlife populations. Additionally, more efficient tracking of zoonotic disease outbreaks will be possible with the establishment of integrated surveillance systems that connect data on human and animal health [38]. Reducing dependence on bushmeat as the main food source and encouraging sustainable hunting methods can help lower the risk of zoonotic infection. The demand for bushmeat and interactions between people and wildlife can be decreased by promoting the creation of alternate sources of income for hunters and assisting with agricultural methods that improve food security. These alternate sources can include activities that give a steady income and lessen dependency on hunting, such as growing cash crops like cocoa, rearing and domesticating desirable animal species, and aquaculture [39]. To reduce direct contact with ticks and diseased wildlife, laws controlling the hunting, trading, and consumption of bushmeat should also be tightened. The risk of zoonotic infections can be considerably decreased by wearing protective gear when hunting and by using safe bushmeat preparation methods. Lastly, to detect and control emerging zoonotic diseases in Ghana, funding for research on tick‐borne pathogens and the creation of diagnostic tools would be essential.

## 2 Conclusion

Consuming bushmeat is a significant aspect of Ghanaian culture and the local economy. However, it also poses an increasing concern to public health since it may spread zoonotic tick‐borne pathogens. The probability of zoonotic infections is increasing along with the frequency of human‐wildlife‐tick contacts due to habitat encroachment and environmental changes. Mitigating the risk of zoonotic tick‐borne pathogens in Ghana requires strengthened public health measures, including enhanced wildlife monitoring, targeted education programs, and stricter regulatory enforcement. These initiatives are essential to safeguard the health of people and animals as the demand for bushmeat rises.

## Author Contributions

**Christopher Nii Laryea Tawiah‐Mensah**: investigation, writing – review and editing. **Danielle Ladzekpo**: investigation, writing – review and editing. **Seth Offei Addo**: conceptualization, writing – original draft, investigation.

## Conflicts of Interest

The authors declare no conflicts of interest.

## Data Availability

No new data were generated for this article.
